# A novel approach to treat peri implantitis with the help of PRF

**DOI:** 10.11604/pamj.2017.27.256.12544

**Published:** 2017-08-07

**Authors:** Rashmi Shah, Hemal Shah, Omkar Shetty, Gaurang Mistry

**Affiliations:** 1 Department of Prosthodontics, DY Patil School of Dentistry, Navi Mumbai, India

**Keywords:** Peri implantitis, PRF, keratinsed mucosa

## Abstract

A new field of biomédical science, known as tissue engineering, applies the principles of biology and engineering to the development of functional substitutes for tissues and organs. Little is known about the influence of Keratinised Mucosa dimension around implants on the soft and hard tissue health The purpose of this case report is to understand the importance of healthy tissue around the implants for long term success and how biomimetic materials like PRF Membrane can be incorporated in treatment plan as an important modality in selected cases.

## Introduction

Replacing missing teeth with dental implants has become a popular procedure among patients and clinicians because of the high survival rate and the predictability of the procedure. Long-term follow-up studies have shown that peri-implant complications are common and that implant survival does not necessarily mean a successful implant. Furthermore, studies have shown that 5% to 11% of all dental implants fail and have to be removed [1, 2]. When a foreign body is placed in bone or soft tissue, an inflammatory reaction inevitably develops. Hence, osseointegration is but a foreign body response to the implant, which according to classic pathology is a chronic inflammatory response and characterized by bone embedding/separation of the implant from the body. The osseointegrated interface remains in a very delicate balance where adverse individual tissue reactions may combine with the foreign body reaction to cause unwanted sequel in form of marginal bone loss or implant failure. The *locus resistentiae minoris* created by the foreign body reaction has resulted in a series of events potentially leading to implant failure. An increased number of procedures with no clear etiology of complications has led to an increase in cases of peri-implant disease. Peri-implant diseases may occur in two forms, peri-implant mucositis and peri-implantitis [3]. *Peri-implant mucositis* is an inflammatory lesion that resides in the soft tissue surrounding a dental implant without signs of bone loss following the initial bone remodeling. In contrast, peri-implantitis also affects the supporting bone, causing progressive bone loss beyond the normal biologic remodeling.

Periodontal implant complications can be biologic, technical and/or esthetic in origin. Prognosis, once disease is already present, will be determined by the ability to restore those characteristics. Diagnosis of peri-implant diseases requires the measurement of probing depth (PD), BOP and clinical attachment level (CAL) and a radiographic examination to further analyze the characteristic and extent of bone loss, if present. Different treatment strategies, often combined, have been proposed, including mechanical debridement, pharmaceutical therapy and surgical procedures (e.g open-flap debridement, smoothing the implant surface and “decontamination” or “detoxification” of the implant surface followed by resective and/ or bone regenerative procedures to correct the anatomical conditions for improving plaque control and for eliminating the pathologic peri-implant pockets). Although nonsurgical treatments are recommended for mucositis lesions, their use has been proven not to be effective in peri-implantitis lesions [4]. The greatest challenge in clinical research is development of bioactive surgical additives, which help to regulate inflammation and increase the speed of healing process [5]. A new field of biomédical science, known as tissue engineering, applies the principles of biology and engineering to the development of functional substitutes for tissues and organs. The triad that constitutes the base of tissue engineering with a reparative objective is formed by the following: matrices or scaffolds, with various presentations (gels, fibrous matrices, permeable membranes), progenitor cells (undifferentiated stem cells, or cells with preliminary differentiations) and growth factors.

Regenerative potential of platelets was intorduced in 1974 and Ross et al were amongst the pioneers who first described a growth factor from platelets. Platelet Rich Plasma (PRP) is a preparation of plasma that contains an increased concentration of platelets compared to whole blood. PRP was introduced to the dental community by Whitman and colleagues, who hypothesized that the activation of platelets and the subsequent release of growth factors would enhance surgical healing [6]. Specific protocols and automated systems for preparing PRP have been developed and commercialized, including Ace, PRGF, PRP-Landesber, Curasan, PCCS, Harvest SmartPReP, Vivostat, Friadent-Schutze, Regen, Fibrinet and Plateltex. Dohan Ehrenfest and colleagues offer an excellent comparison between these traditional systems [7]. While these widely used methods tend to be comparatively expensive and complex and often use 50mL or more of blood, simpler and less expensive methods that require less than 10mL of blood have also been developed in the last few years. Leukocytes and Platelet-rich fibrin (L-PRF), developed in France by Choukroun et al (2001), is a second generation platelet concentrate widely used to accelerate soft and hard tissue healing. Its advantages over the better known platelet-rich plasma (PRP) include ease of preparation/application, minimal expense and lack of biochemical modification (no bovine thrombin or anticoagulant is required). L-PRF is a strictly autologous fibrin matrix containing a large quantity of platelet and leukocyte cytokines. The purpose of this case report is to understand the importance of healthy tissue around the implants for long term success and how biomimetic materials like PRF Membrane can be incorporated in treatment plan as an important modality in selected cases as this one.

## Patient and observation

**Figure 1:**
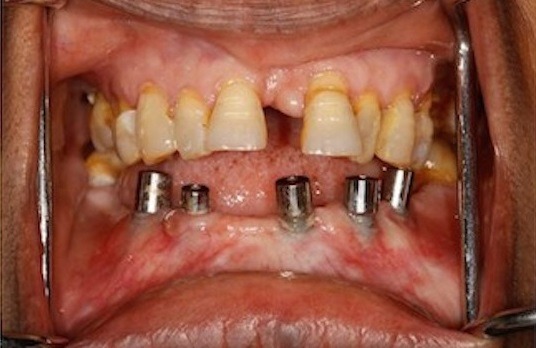
Pre operative

47 yr old female patient reported to the Department of Prosthodontics, six months after the surgery, for restoration of five implants placed in the mid-symphysis region in the lower jaw. On clinical evaluation following observations were made, in the implants probing depth was less than 3mm, there was no mobility of the implants, no bleeding on probing or any pus discharge, no active pockets were present. The width of attached Gingiva was < 2mm along with soft tissue dehiscence, exposing 3 to 4 threads on all the three implants in the third quadrant. In the fourth quadrant there was attached gingiva of about 3mm present on mesial implant but the distal implant showed less < 2mm of width of attached gingiva (Figure 1).

**L-prf procedure**: Immediately prior to surgery, 10ml of blood was drawn from the patient by venipuncture of the antecubital vein and collected in a sterile glass test tube without any anticoagulant. The blood was quickly collected and the tubes were immediately centrifuged at 2,700 rpm for 12 minutes [8] using a centrifuge machine, at room temperature. Because of differential densities, centrifugation resulted in separation of the following three basic fractions: red blood cells at the bottom, acellular plasma on the surface and an L-PRF clot formed in the middle part of the tube. After centrifugation, the L-PRF clots were removed from the tubes using sterile tweezers and placed on sterile woven gauze. Clots were emptied from their serum by compressing them between two pieces of woven gauze. L-PRF membranes were prepared by compressing clots between two pieces of woven gauze. In the third quadrant, partial thickness incision was made extending horizontally 2 to 3mm beyond the mesial and distal most implants and the Vertical incisions were extending into the vestibule. Partial thickness flap was raised and the amount of implant exposed was now clearly evident. The contaminated surface of the implant was smoothened off with a fine grid polishing diamond and rubber polisher (implantoplasty) and the surface disinfected with chlorhexidine 0.12% [9] to remove all bacteria deposits, facilitate soft tissue re-accommodation and limit and minimize future plaque deposition that would reinitiate the disease episode.

**Figure 2:**
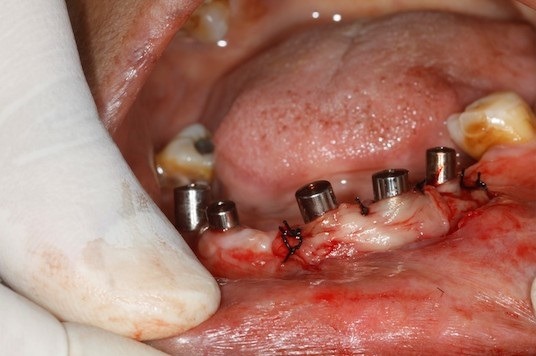
PRF membranes secured with sling sutures

**Figure 3:**
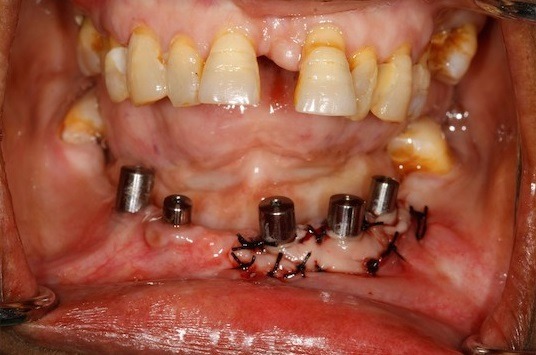
Vestibuloplasty with PRF membrane in 3^rd^ quadrant

**Figure 4:**
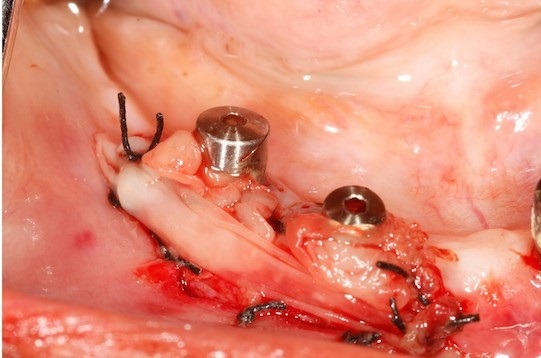
Vestibuloplasty with PRF membrane in 4^th^ quadrant

**Figure 5:**
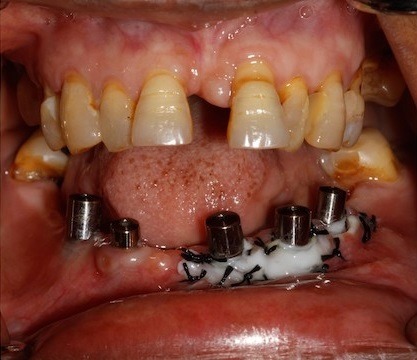
Day 1 after surgery, PRF membrane whitish in colour

**Figure 6:**
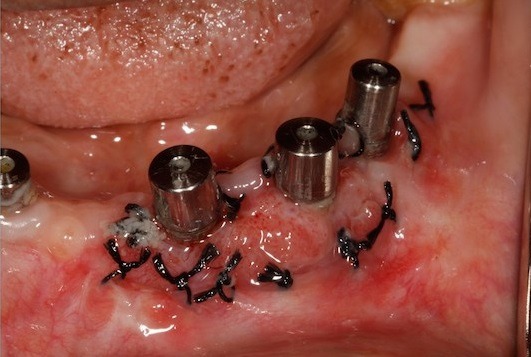
After 8 days, new immature blood vessels seen

**Figure 7:**
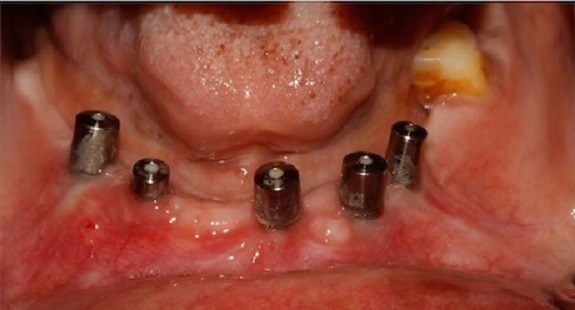
After 4 week, sufficient coverage of exposed implant surface

The 2 PRF membrane's are placed over lapping each other to cover all the three implants and are secured around the gingival former with the help of sling sutures (Figure 2). The coronal end of the partial thickness flap is positioned apically and sutured along with the apical end of the PRF membrane, to the underlying periostium, using 4-0 silk (Figure 3) (vestibuloplasty with PRF membrane). The PRF membrane was kept exposed to the oral. In the fourth quadrant, surgery was performed after sufficient healing was seen in third quadrant. PRF membrane was prepared in similar fashion. Sulcular incison was taken and two short vertical releasing incisions were performed. Thereafter a split-thickness mucosal flap was prepared in the vestibule, whose coronal margins were sutured apically with the periosteum. PRF membrane was then placed over the exposed connective tissue bed and secured around the gingival former with stabilizing suture and sling suture respectively (Figure 4) (vestibuloplasty with PRF membrane). In both the quadrants, on day 1 after the surgery, the surface of the PRF membrane appeared whitish in colour (Figure 5) but by day 8, neo-angiogenesis had set it and many new immature blood vessels were seen (Figure 6). By the end of 4 weeks (Figure 7) the change in the tissue biotype from thin to thick, sufficient coverage of the exposed implant surfaces was achieved, there was evident increase in the vestibular depth and on pulling of the lower lips, no blanching of the marginal gingiva was visible. Significant difference was observed between the pre-operative condition and the post-operative condition.

## Discussion

Historically, an increased zone of keratinized tissue was presumed desirable tor the following reasons: provides a resistant barrier to plaque induced inflammation; replaces unkeratinized margins to prevent recession; deepens vestibules to provide better access for tooth brushing; dissipates functional and masticatory stress placed on the gingival margin of a restoration; and improves esthetics, patient comfort and ease of hygiene. Little is known about the influence of Keratinised Mucosa dimension around implants on the soft and hard tissue health. Comparatively few studies are available examining the relationship between the width of Keratinised Mucosa and the health of periimplant tissues. Block & Kent (1990) reported that in the posterior mandible, the presence of Keratinised Mucosa was strongly correlated with optimal mucosal health and the lack of Keratinised Mucosa was in association with a crestal bone loss of 2mm or more. Likewise, an experimental study in monkeys examined the influence of bacterial plaque on attachment loss around implants placed in areas with or without Keratinised Mucosa (Warrer et al. 1995). It was found that ligated implants without keratinized mucosa demonstrated significantly more recession and slightly more attachment loss than implants with keratinized mucosa. Thus the Keratinised Mucosa thickness and width around dental implants affects both the clinical and the immunological parameters at these sites [10]. According to the recently proposed guidelines for the treatment of peri-implant disease [11], in this case with no bleeding on probing, no pockets, probing depth less than 3mm, no pus discharge and also when the amount of keratinized gingiva is less than 2mm with soft tissue dehesence.

The ideal treatment would be soft tissue grafting procedures. Basically, two different peri-implant soft tissue augmentation methods can be applied [12]: enlargement of Keratinised Mucosa width by means of an apically positioned flap/vestibuloplasty (in combination with a Free Gingival Graft); gain of soft tissue volume using a sub-epithelial Connective Tissue Graft or soft tissue replacement graft. The ideal treatment plan in this cases of a thin mucosa and a minimal width or lack of peri-implant Keratinised Mucosa, in combination with a soft tissue recession to the extent that the rough implant surface which was visible, a two-stage procedure should have be performed: first a sub-epithelial connective tissue graft in combination with a coronally advanced flap in order to cover the dehiscence; and second, after a healing period of 3 months, a vestibuloplasty in combination with a free gingival graft to create an adequate peri-implant cuff of Keratinised Mucosa. The clinical procedure of sub-epithelial connective tissue graft harvesting from the palate is often characterized by the remarkable challenge of obtaining the largest volume of tissue possible on one side while minimizing post-operative pain and reducing the risk of complications on the other side. The blood vessels that supply the lateral palate region. The greater palatine artery, emerges from the greater palatine foramen and extends along the lateral palate in an anterior direction. Due to its size, injury to the greater palatine artery, particularly in its distal segment, can be expected to result in massive bleeding. It is therefore crucial to take precautions to prevent damage to the artery when subepithelial connective tissue grafts are harvested from the palate. In this patient the amount of tissue available would not have sufficed the amount of surface area of three implants to be covered. Therefore, PRF membrane was chosen as an substitute for soft tissue grafting. PRF is a complex regenerative scaffold that consists of a homogenous 3-dimenzional organization of fibrin matrix polymerized in a tetra-molecular structure, with incorporation of platelets, leucocytes, cytokines, glycosaminoglycans and circulating stem cells.

Fibrin is activated form of fibrinogen present in blood plasma and α-granules of platelets. Soluble fibrinogen is transformed in insoluble polymerized fibrin matrix during hemostasis [13]. The fibrin matrix present in PRF is flexible, elastic, stable and very strong [14]. L-PRF is an optimized blood clot with stronger fibrin architecture and mechanical properties than a natural blood clot or a PRP gel. In comparison of a natural blood clot or a PRP gel, a L-PRF membrane is a solid material easier to handle and to position in the defects and this is also a considerable advantage. While other membranes are considered as foreign bodies by the host tissues and interfere with the natural tissue healing process, a L-PRF membrane is as natural as the host tissue: it is a blood clot prepared in an optimized form. The property of slow release of growth factors and matrix proteins from the L-PRF membrane promotes 2 specific biological mechanisms during coverage: impregnation and induction [15]. First the surface is impregnated with blood proteins, which are the first biological links between the surface and a new attachment: this is an old validated concept in periodontal surgery [16]. Second, contrarily to PRP gels which gives away immediate release of growth factors, the slow release of molecules from the L-PRF membrane lasts several days [17, 18] and thus is long enough to trigger a cell induction phenomenon [19]. The growth factors stimulate cell proliferation, neovessels develop within the fibrin matrix, the periosteum is stimulated, the gingival fibroblasts migrate within the fibrin matrix and slowly remodel it, while the membrane guides surface epithelialization if needed: this matrix offers a new scheme of development to the natural tissue. The short term result is a quick wound closure and healing [20], with the reduction of postsurgical pain and edema. The long term result is not only a stable covering, but also a thicker stable gingiva.

## Conclusion

The use of PRF in implant applications is in its infancy. The potential clinical implications for this inexpensive and autologous material are promising. The final goal should be to develop an appropriate soft tissue substitute, suitable for all different clinical situations and by doing so, eliminate the need for donor site morbidity and its associated complications. The ease of fabrication of PRF should encourage more and more smaller practices to incorporate it in their practises. This should allow long term longitudinal, multicentre studies to be conducted. A systemic review of these studies should help us gather data sufficient enough to develop evidence based consensus on various questions like, mode of fabrication, techniques of clinical application etc.

## Competing interests

The authors declare no competing interest.
